# 3D-Printed PLA/PETG Sterilizable Static/Dynamic Modular External Finger Fracture Fixator

**DOI:** 10.3390/bioengineering13090969

**Published:** 2026-08-24

**Authors:** Xavier Soong, Alyssa Rothman, Eric Tolo, Maximillian Soong, N. George Kasparyan

**Affiliations:** 1 Boston University Academy, Boston, MA 02215, USA; 2 Department of Orthopedic Surgery, Lahey Clinic, Burlington, MA 01805, USA; 3 Department of Orthopedic Surgery, Chobanian & Avedisian School of Medicine, Boston University, Boston, MA 02118, USA; 4 Department of Orthopedic Surgery, University of Massachusetts Chan Medical School, Lahey Campus, Burlington, MA 01805, USA

**Keywords:** 3D-printed, polylactic acid, polyethylene terephthalate glycol, finger fracture, sterile, surgery

## Abstract

Finger fractures are common and potentially disabling. Certain complex injuries, particularly with small fragments and/or open wounds, are not manageable with conventional orthopedic hardware. We created 3D-printed finger fracture fixators using polylactic acid (PLA) and polyethylene terephthalate glycol (PETG). We tested flexion stiffness and lateral stiffness, before and after sterilization for surgical use, and compared them against standard 316L surgical stainless-steel (SSS) wires. Because PLA and PETG are compromised at high temperatures, these were sterilized using hydrogen peroxide gas plasma, while the SSS wires were autoclaved. The PLA fixators demonstrated greater flexion stiffness, and equivalent lateral stiffness, to the SSS wires, both before and after sterilization. The PETG fixators demonstrated inferior flexion stiffness and lateral stiffness to the SSS wires, and these properties worsened with sterilization. The PLA fixator was then applied to a cadaveric hand in static and dynamic configurations, and demonstrated excellent radiolucency for visualization of bone alignment and healing. This novel PLA device secures numerous surgical stainless-steel wires, maintains greater flexion stiffness and equivalent lateral stiffness compared to the wires even after sterilization, allows for static and dynamic fixation with compact, lightweight, and modular radiolucent components, and is both low-cost and customizable on demand, which may benefit under-resourced communities.

## 1. Introduction

Finger fractures are common, with over 100,000 cases reported in the United States annually, and may result in considerable disability [1]. Some complex finger fractures, particularly with small fragments and/or open wounds, are not amenable to standard treatment with braces, casts, and conventional orthopedic hardware including wires, screws, and plates, typically made from 316L surgical stainless-steel or Ti-6Al-4V titanium alloy [2]. Such fractures in larger bones such as the femur and tibia can be secured with external fixators, which have been scaled down for the hand, yet they remain bulky and heavy, consisting of multiple bars and clamps and requiring large trays of equipment. Most current external fixators are static, lacking an option for dynamic fixation to allow joint motion, which is important to minimize contractures that often develop during healing. They also have large radio-opaque clamps, which obscure radiographic visualization of bone alignment and bone healing. Furthermore, current fixators are costly and thus limited in under-resourced communities.

Hand surgeons have therefore resorted to improvised rigid bar devices to secure surgical wires in fingers. These bar devices are readily available in the hospital as sterile pre-packaged items, such as long hypodermic needle caps [3,4,5], insulin syringes [2,6,7], or plastic tubes reinforced with orthopedic polymethylmethacrylate bone cement [8,9]. Although feasible, technical problems with these devices include: drilling through curved surfaces of these cylindrical items without slipping off target; maintaining fracture alignment and safety with sharp wires despite the force required to drill through both curved surfaces of the hollow structure; generation of fine plastic debris that infiltrates the wound; and difficulty or inability to reposition or replace the device after fixation.

3D-printing has been increasingly utilized for orthopedic applications, including the creation of patient-specific customized orthotics and prosthetics [10,11,12], fixation plates [13,14], as well as surgical planning guides and models [15,16]. 3D-printed external fixators have been developed for large bones and have tested favorably against conventional devices when produced at 100% infill [11,17,18,19]. However, smaller devices for finger fractures are lacking.

Importantly, 3D-printed devices for finger fracture fixation must maintain strength throughout the sterilization process in order to provide stability during application and bone healing. This requires specific machines and protocols, as the high temperatures of steam autoclaves used for most surgical instruments, ranging from 121 °C to 134 °C, are not compatible with commonly used 3D-printing materials, including plastics such as polylactic acid (PLA) and polyethylene terephthalate glycol (PETG), which have glass transition temperatures of approximately 60 °C and 80 °C, respectively. Previous studies have shown that sterilization with hydrogen peroxide gas plasma (HPGP), at temperatures ranging from 47 °C to 56 °C, does not significantly affect PLA with regard to morphology, tensile strength, elongation, and Young’s modulus [20,21,22]. However, specific testing of flexion stiffness and lateral stiffness of a 3D-printed finger fracture fixator device has not been reported.

Our purpose was to create and validate a finger fracture fixator that is:

- Readily able to hold numerous surgical wires in stable position;
- Comparably stiff to the surgical wires it holds;
- Sterilizable with HPGP for surgical use without compromising strength;
- Capable of both static and dynamic fixation to allow joint motion;
- Appropriately compact and lightweight for use on fingers;
- Modular to allow versatile application, repositioning, replacement, expansion;
- Radiolucent to facilitate radiographs of bone alignment and bone healing;
- Low-cost to produce and customizable on demand.

## 2. Materials and Methods

### 2.1. Fixator Design and Production

**Figure 1:**
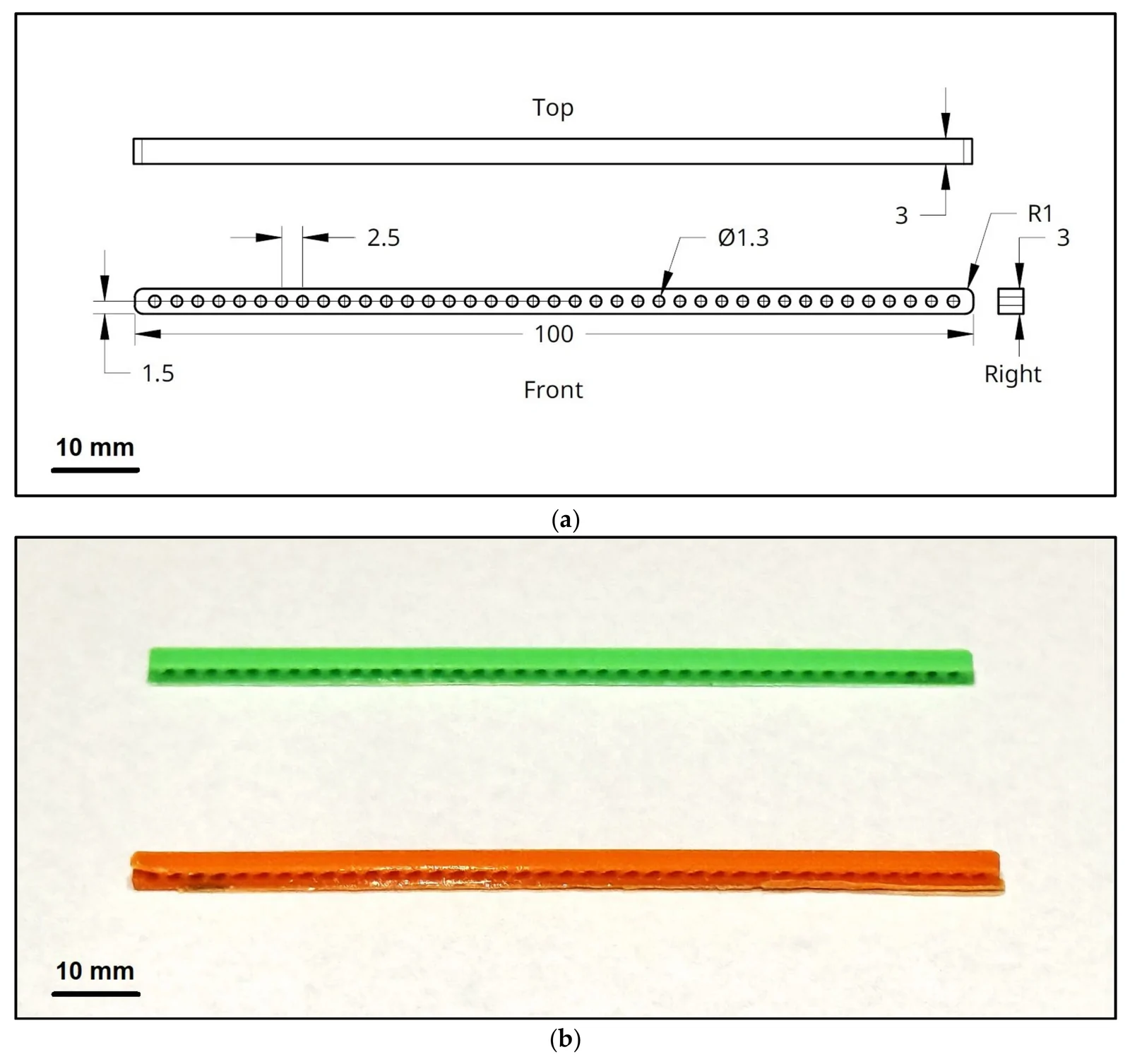
Finger fracture fixator: (**a**) computer-aided design drawing, measurements are in mm, (**b**) 3D-printed PLA (green) and PETG (orange) fixators.

We designed our finger fracture fixator using Onshape (online platform, https://www.onshape.com/en/, accessed November 2025) computer-aided design software (Figure 1a). The device measures 3 × 3 mm in cross section, and is 10 cm in length. Along the device are 39 holes, each 1.3 mm in diameter, centered 2.5 mm apart. We prepared for 3D-printing of the device by using UltiMaker Cura (version 5.11.0) model slicing software. We then produced fixators using the Sidewinder X2 3D-printer (Artillery, Shenzhen, China), with the nozzle heated to 210 °C and pointing perpendicular to the hole paths, which lie parallel across the print bed, at a printing speed of 55 mm/s, running predominantly along the major axis of the fixator, with a layer height of 0.12 mm and 100% infill. Filaments used were Panchroma Neon Green PLA (Polymaker, Changshu, China) and Orange PETG (Hatchbox, Rowland Heights, CA, USA) (Figure 1b). Three sample devices of each material were produced and tested along with three 316L surgical stainless-steel (SSS) wires measuring 10 cm in length with 1.1 mm diameter (MicroAire Surgical Instruments, Charlottesville, VA, USA), which are the most commonly used wires for finger fracture fixation. We tested each fixator against SSS in order to demonstrate that the fixator is not the weakest component of the wire/fixator construct, and is therefore appropriate for this usage.

### 2.2. Stiffness Testing, Sterilization, and Application Procedures

**Figure 2:**
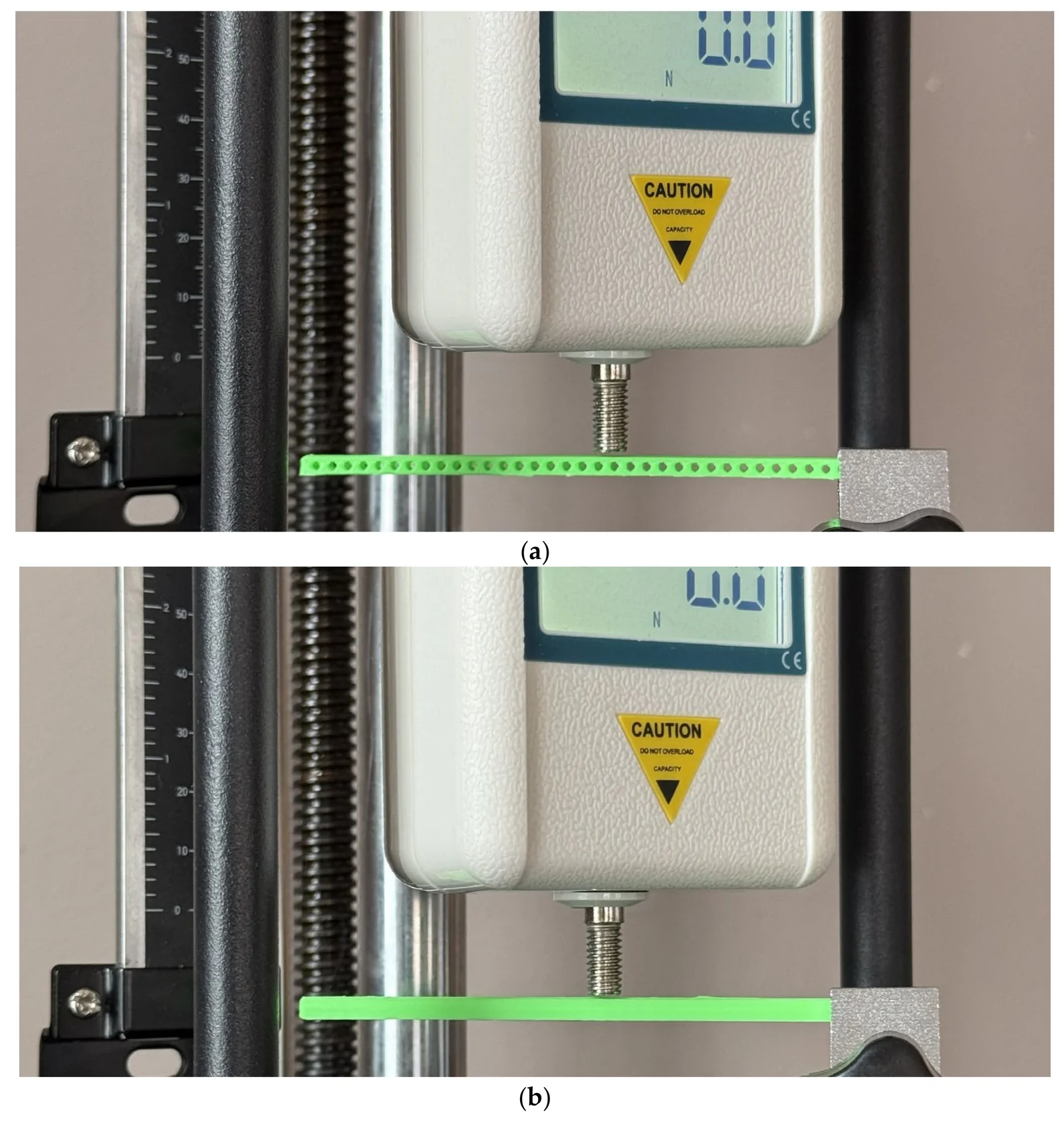
Stiffness testing: (**a**) flexion stiffness, with transverse holes visible, (**b**) lateral stiffness, with transverse holes not visible.

To test flexion stiffness, we secured each PLA or PETG fixator horizontally in a force gauge stand (including a load cell with ±0.5% accuracy and a 1 kHz sampling rate), flush with the end of the mounting clamp, with the wire holes oriented horizontally (Figure 2a). We then applied a 5.0-newton force at the midpoint of the fixator, while measuring displacement with the digital meter. To test lateral stiffness, we secured each PLA or PETG fixator with the wire holes oriented vertically (i.e., the fixators were rotated 90 degrees along their longitudinal axes) (Figure 2b). We again applied a 5.0-newton force at the midpoint of the fixator, while measuring displacement. The SSS wires were tested similarly, although stiffness values were accepted to represent both flexion stiffness and lateral stiffness, as the wires are rotationally symmetric.

We then sterilized the PLA and PETG fixators in a STERRAD NX hydrogen peroxide gas plasma (HPGP) sterilizer (Advanced Sterilization Products, Irvine, CA, USA) at 56 °C for 54 min, and the SSS wires in an AMSCO 400 autoclave (STERIS, Mentor, OH, USA) at 132 °C for 4 min, per the respective standard protocols. After sterilization, we repeated the flexion stiffness and lateral stiffness testing protocols as above using the sterilized devices. We calculated stiffness in N/mm using the displacement data.

We then applied the 3D-printed fixator with greater stiffness to a cadaveric human hand in static and dynamic configurations, securing the fixator to the index finger by drilling wires transversely across the bones with a System 8 cordless drill (Stryker, Kalamazaoo, MI, USA, while obtaining fluoroscopic images on a Fluoroscan InSight mini C-arm (Hologic, Marlborough, MA, USA). These images were then measured for luminance using Microsoft Paint (Version 22H2).

### 2.3. Statistical Analysis

Data were analyzed using Microsoft Excel (Version 2607). Means and standard deviations were calculated. Comparisons were performed using a two-tailed *t*-test, α = 0.05.

## 3. Results

### 3.1. Flexion Stiffness (Figure 3)

Before sterilization, the PLA fixators demonstrated greater flexion stiffness than the SSS wires (1.37 vs. 1.15 N/mm, *p* < 0.05), while the PETG fixators demonstrated less flexion stiffness than the SSS wires (0.89 vs. 1.15 N/mm, *p* < 0.01). Sterilization with HPGP did not significantly affect flexion stiffness for PLA, although PETG was significantly weakened (0.80 vs. 0.89 N/mm, *p* < 0.05), and the SSS wires were not significantly affected by autoclaving. Thus, after sterilization, the PLA fixators again demonstrated greater flexion stiffness than the SSS wires (1.32 vs. 1.14 N/mm, *p* < 0.05), while the PETG fixators again demonstrated less flexion stiffness than the SSS wires (0.80 vs. 1.14 N/mm, *p* < 0.001).

**Figure 3:**
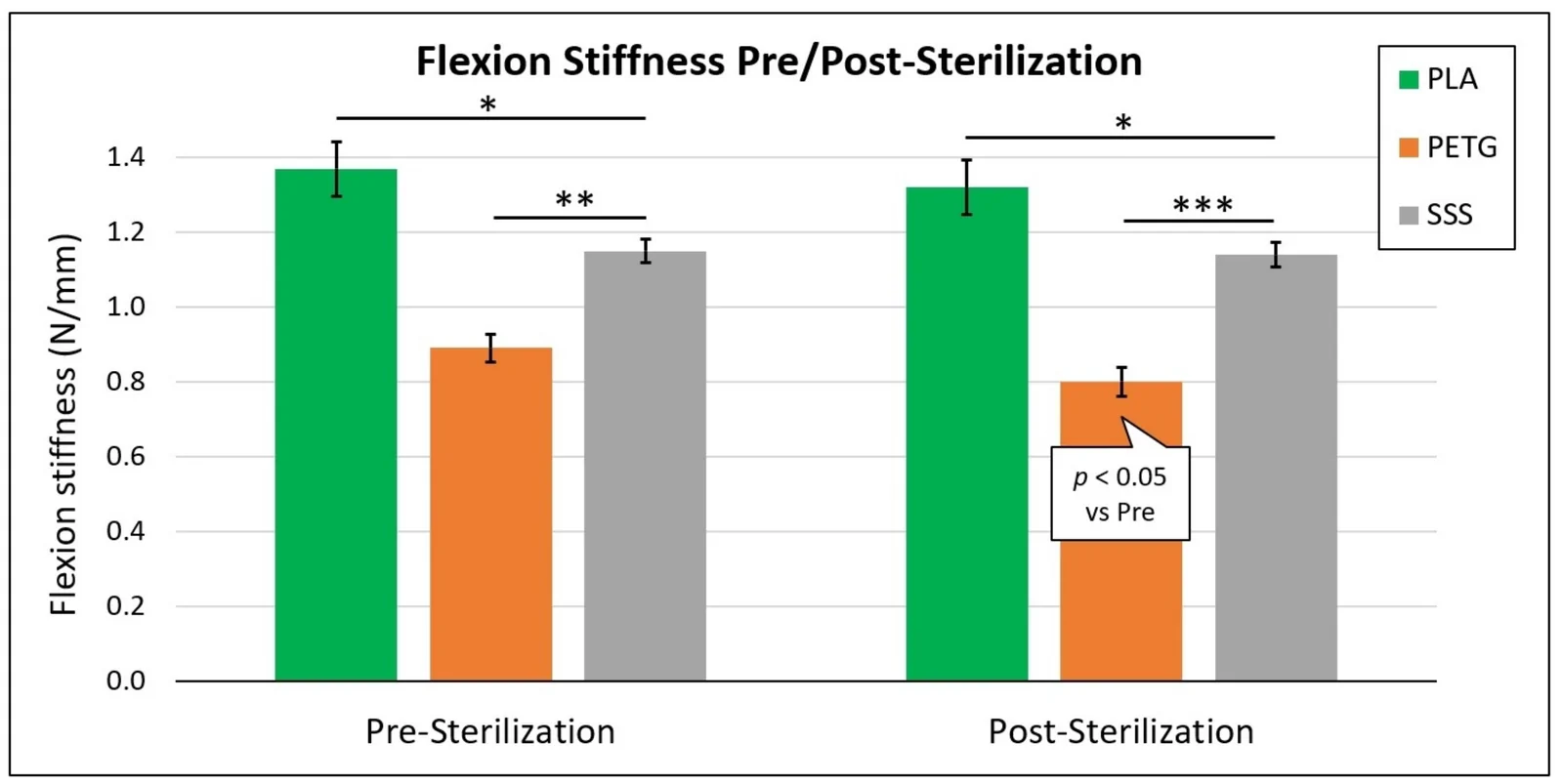
Flexion stiffness before and after sterilization. PLA, polylactic acid; PETG, polyethylene terephthalate glycol; SSS, surgical stainless-steel. Bars demonstrate means. Error bars demonstrate standard deviations. Significant differences include: *, *p* < 0.05; **, *p* < 0.01; ***, *p* < 0.001.

**Table 1:** Flexion stiffness and lateral stiffness of PLA, PETG, and SSS, before and after sterilization.

Parameter	Material	Stiffness (N/mm)	Comparison (*p* Value)
Flexion stiffness, pre-sterilization	PLA	1.37 ± 0.070	<0.05 vs. SSS
	PETG	0.89 ± 0.040	<0.01 vs. SSS
	SSS	1.15 + 0.038	
Flexion stiffness, post-sterilization	PLA	1.32 ± 0.075	<0.05 vs. SSS, NS vs. pre-sterile
	PETG	0.80 ± 0.035	<0.001 vs. SSS, <0.05 vs. pre-sterile
	SSS	1.14 ± 0.025	NS vs. pre-sterile
Lateral stiffness, pre-sterilization	PLA	1.20 ± 0.057	NS vs. SSS, <0.05 vs. flexion
	PETG	0.71 ± 0.026	<0.001 vs. SSS, <0.01 vs. flexion
	SSS	1.15 ± 0.038	
Lateral stiffness, post-sterilization	PLA	1.16 ± 0.040	NS vs. SSS, NS vs. pre-sterile, <0.05 vs. flexion
	PETG	0.63 ± 0.031	<0.001 vs. SSS, <0.05 vs. pre-sterile, <0.01 vs. flexion
	SSS	1.14 ± 0.025	NS vs. pre-sterile

PLA, polylactic acid; PETG, polyethylene terephthalate glycol; SSS, surgical stainless-steel. Values presented are mean ± standard deviation; NS, non-significant.

### 3.2. Lateral Stiffness (Figure 4)

Before sterilization, the PLA fixators demonstrated no significant difference in lateral stiffness compared to the SSS wires, while the PETG fixators demonstrated less lateral stiffness than the SSS wires (0.71 vs. 1.15 N/mm, *p* < 0.001). Sterilization with HPGP did not significantly affect lateral stiffness for PLA, although PETG was significantly weakened (0.63 vs. 0.71 N/mm, *p* < 0.05), and the SSS wires were not significantly affected by autoclaving. Thus, after sterilization, the PLA fixators demonstrated no significant difference in lateral stiffness compared to the SSS wires, while the PETG fixators again demonstrated less lateral stiffness than the SSS wires (0.63 vs. 1.14 N/mm, *p* < 0.001).

**Figure 4:**
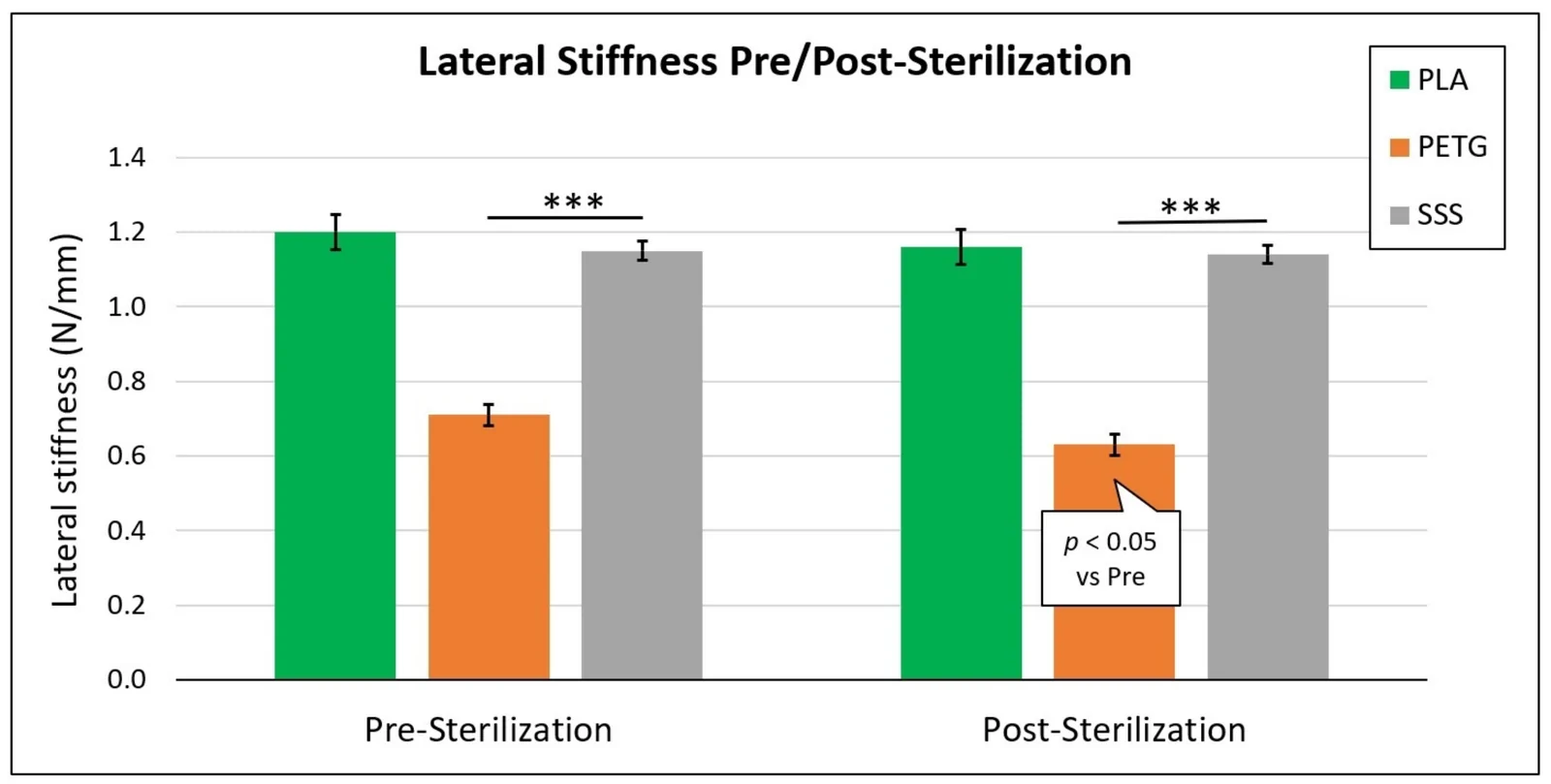
Lateral stiffness before and after sterilization. PLA, polylactic acid; PETG, polyethylene terephthalate glycol; SSS, surgical stainless-steel. Bars demonstrate means. Error bars demonstrate standard deviations. Significant differences include: ***, *p* < 0.001.

### 3.3. Flexion Stiffness Versus Lateral Stiffness (Table 1)

The PLA fixators demonstrated significantly greater flexion stiffness than lateral stiffness, both before sterilization (1.37 vs. 1.20 N/mm, *p* < 0.05) and after sterilization (1.32 vs. 1.16 N/mm, *p* < 0.05). Similarly, the PETG fixators demonstrated significantly greater flexion stiffness than lateral stiffness, both before sterilization (0.89 vs. 0.71 N/mm, *p* < 0.01) and after sterilization (0.80 vs. 0.63 N/mm, *p* < 0.01).

### 3.4. Cadaver Application

**Figure 5:**
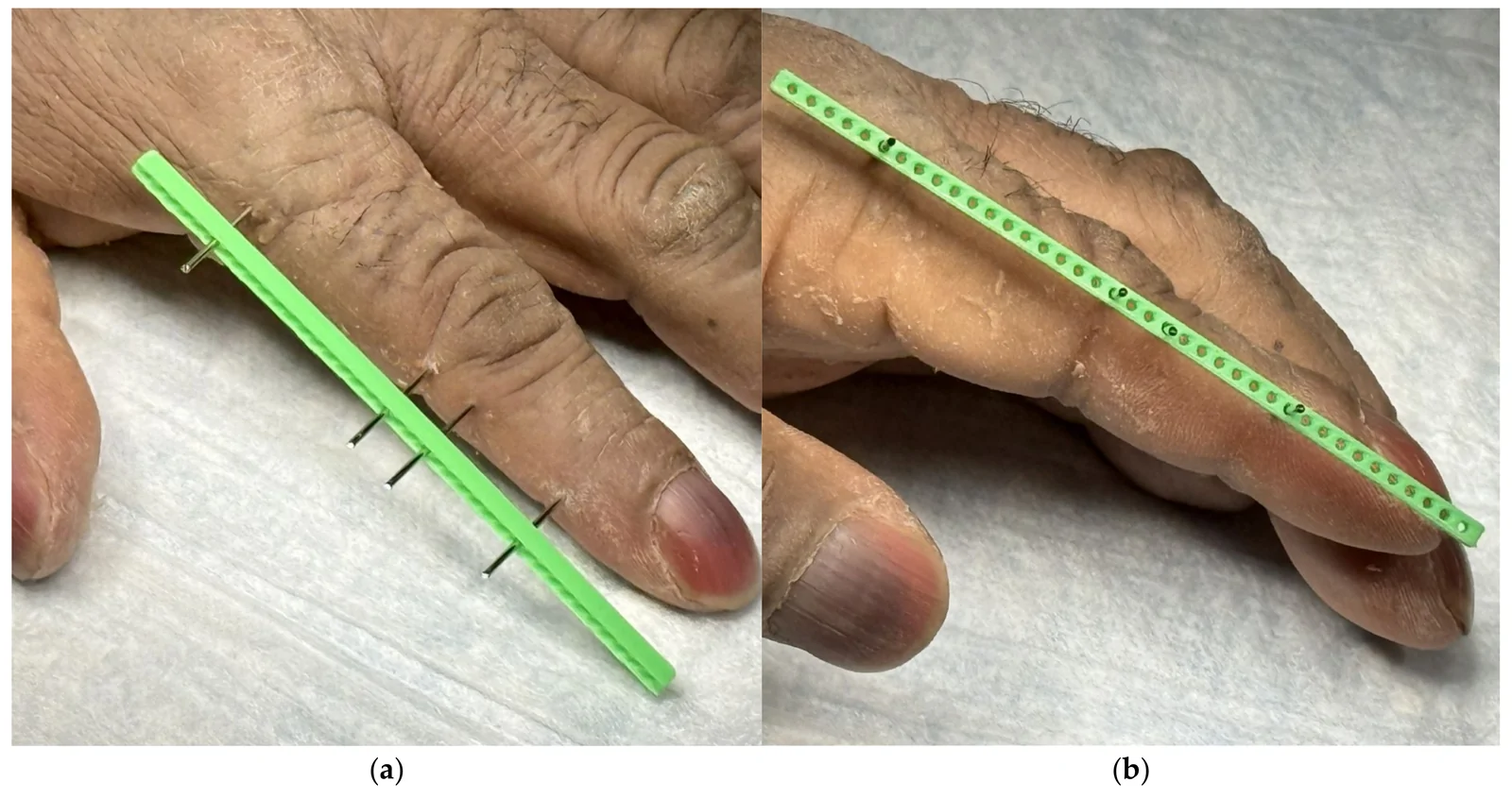
PLA fixator applied to cadaver finger in static configuration: (**a**) dorsal view, (**b**) lateral view.

**Figure 6:**
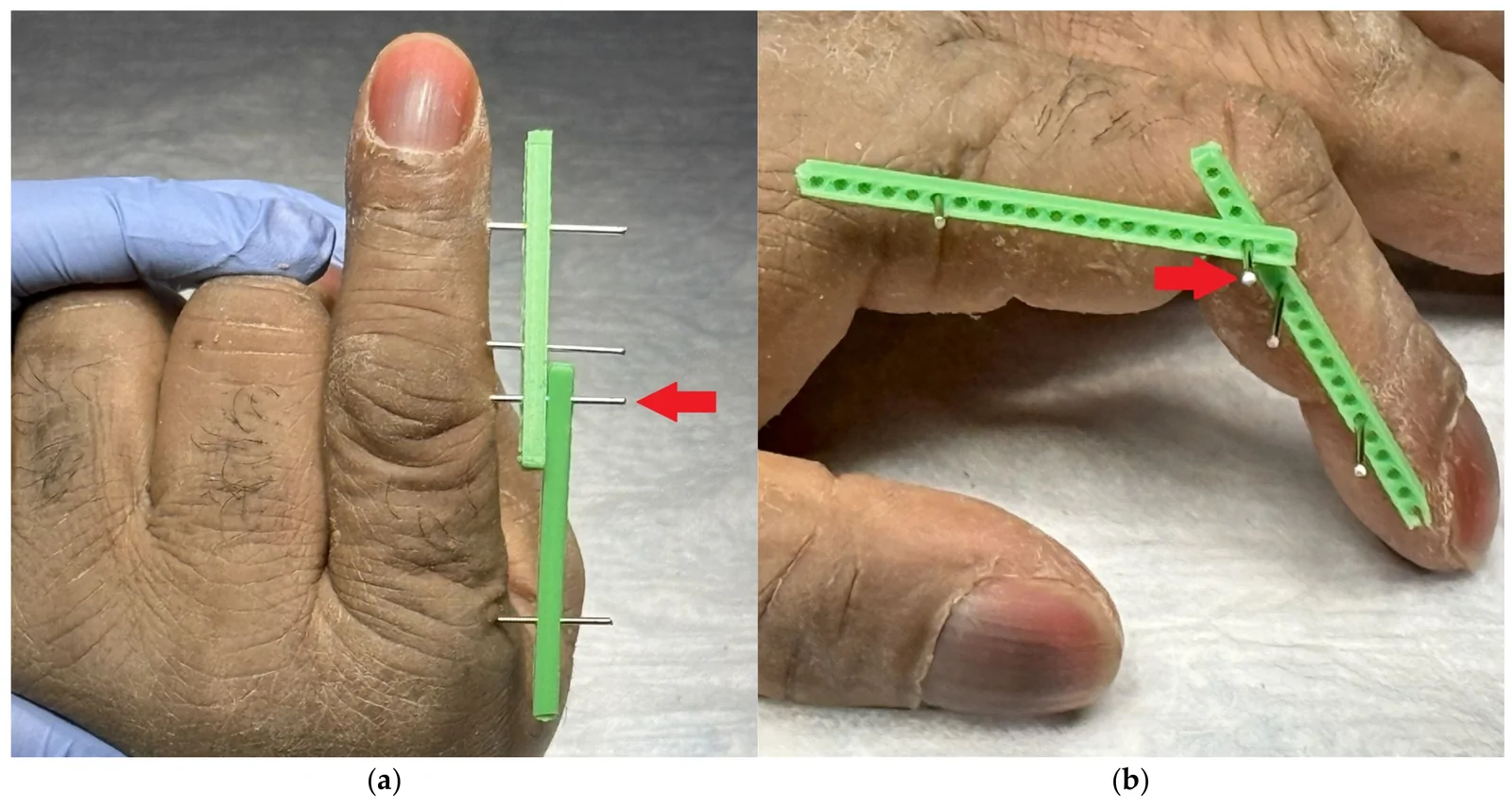
PLA fixator applied to cadaver finger in dynamic configuration: (**a**) dorsal view, (**b**) lateral view. Red arrow indicates wire placed at axis of rotation (proximal interphalangeal joint).

**Figure 7:**
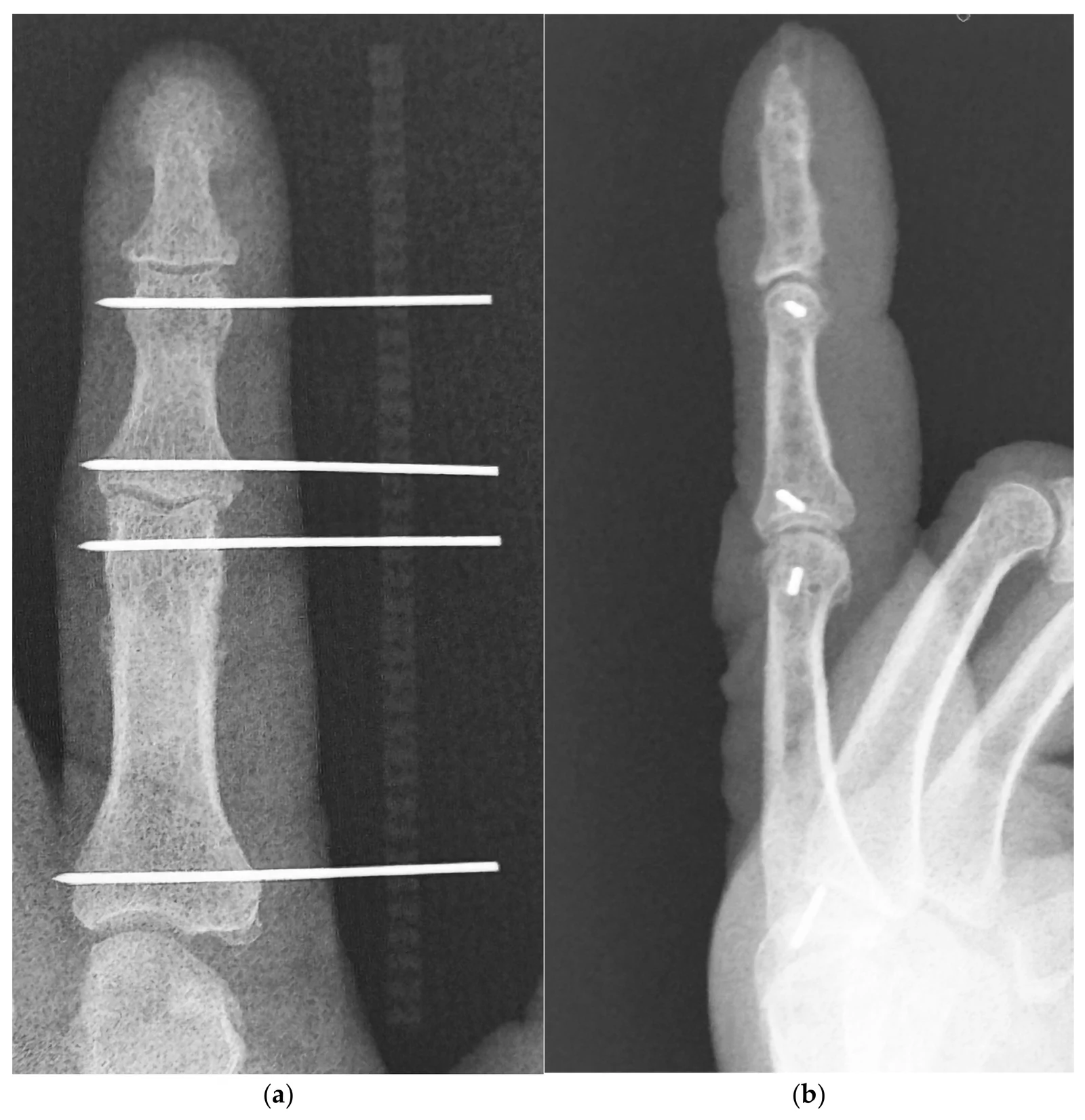
Fluoroscopic images of PLA fixator applied to cadaver finger: (**a**) coronal view, (**b**) lateral view.

The PLA fixator was readily applied to the cadaver hand in static configuration by drilling wires through the holes and into the index finger (Figure 5). By cutting the fixator in half and connecting both segments on a wire placed at an axis of rotation (the proximal interphalangeal joint), dynamic fixation was achieved, allowing joint motion (Figure 6 and Supplementary Video S1). Fluoroscopy demonstrated excellent radiolucency of the fixator, with luminance of 38% compared to the adjacent bone, and 28% compared to the adjacent SSS wire, allowing unobstructed visualization of all finger bones (Figure 7).

## 4. Discussion

This 3D-printed PLA finger fracture fixator provided greater flexion stiffness and equivalent lateral stiffness when compared to standard 316L surgical stainless-steel 1.1 mm wires, both before and after sterilization with hydrogen peroxide gas plasma or an autoclave, respectively. PETG had less flexion stiffness and lateral stiffness than the SSS wires, and worsened after HPGP sterilization. Therefore, the PLA fixator is suitable for the surgical treatment of finger fractures, and is superior to the PETG fixator, which should not be used for this purpose.

An important finding of this study is that HPGP sterilization is a viable option for this surgical application of PLA, without compromising stiffness relative to SSS wires, or risking failures which might occur in a hospital autoclave. Although the melting temperature of PLA is approximately 165 °C, the glass transition temperature is approximately 55–65 °C, at which the material becomes soft and at risk of deformation. For PETG, these values are 245 °C and 80–85 °C, respectively. Therefore, neither material is suitable for autoclave temperatures which range from 121 °C to 134 °C. While both materials may therefore potentially be suitable for sterilization with HPGP at temperatures which range from 47 °C to 56 °C, our testing demonstrated that only PLA maintained flexion stiffness and lateral stiffness, while PETG was significantly weakened. PLA and PETG were selected for this study as they are among the most widely used materials due to their low cost and ease of use. Although polycarbonate (PC) and polyether ether ketone (PEEK) have higher glass transition temperatures that are compatible with an autoclave, they are challenging to print with due to warping potential and moisture-related bubbling. Furthermore, PC releases toxic fumes at printing temperatures and PEEK costs up to 30 times more than PLA.

Another finding is that both the PLA and PETG 3D-printed fixators had greater flexion stiffness than lateral stiffness. This is likely due to the configuration of the holes, which are aligned transversely along the fixator, thereby leaving structural gaps in the lateral walls. In clinical use, flexion stiffness is most important as the injured finger must flex repetitively during healing and rehabilitation, in order to minimize joint contracture, whereas lateral forces are less frequently encountered, such as in accidental circumstances. Even so, the lateral stiffness of the PLA fixator was still equivalent to that of the SSS wire, and therefore not a contraindication to its use.

This PLA fixator has advantages over improvised cylindrical plastic bar devices, with regard to ease of drilling and avoidance of generating debris, and is capable of both static and dynamic fixation. Its modular design allows versatility in application using different lengths and configurations, in order to facilitate repositioning, replacement, and expansion to include other bones and joints. Although we demonstrated the most basic uniplanar, one-sided position of the fixator, its small size allows for additional fixators to secure the same wires on both sides of the bone in parallel for increased stiffness, or double-stacked on the same side of the bone, or in a combined orthogonal biplanar arrangement (e.g., simultaneous dorsal and lateral fixators). This PLA fixator is also radiolucent to facilitate visualization of bone alignment and bone healing. Weighing less than 1 g and costing less than 3 cents for PLA material (separate from the initial cost of the 3D-printer and subsequent costs of labor, sterile processing, etc.), this device is compact, lightweight, and inexpensive, warranting further investigation toward clinical use, and could be particularly beneficial for under-resourced communities.

This study has some limitations. It is well-known that 3D-printing is affected by temperature, humidity, filament hydration, nozzle clogging, etc., which we did not tightly regulate, although our dedicated hospital teaching cadaver laboratory has a controlled climate at approximately 20 °C and 45% relative humidity. Dimensional tolerances may also vary, and are critical to the function of the fixator, as the holes were designed to be only 1.3 mm in diameter, allowing insertion of the 1.1 mm surgical wires with minimal “toggling” (i.e., angulation of the wires within the holes). We therefore recommend that practitioners implementing this design perform preclinical testing of the fixators created with their specific 3D-printers, settings, and materials.

The stiffness testing protocol included only a 5.0-newton force, which approximates the average force from a flexor tendon during active finger flexion [23]. Other magnitudes of force, up to and including the point of fatigue or catastrophic failure, should be tested to determine the limits of the device and a potential margin of safety. Finger fracture fixators must also withstand many repetitive forces during healing and rehabilitation, and commercial fixation devices are typically tested through thousands of cycles. Even with minimal usage, PLA still degrades with time, and thus repeated measurements at later timepoints could be taken, although finger fracture fixators are typically used for under two months, and modularity allows substitution with a new device if needed. Electron microscopy could help reveal material changes from sterilization, fatigue, and time, all of which might affect stiffness. Furthermore, for dynamic configurations, frictional force at the axis of rotation should also be determined, and ideally should be minimized with optimal hole size tolerances, without compromising stability.

This fixator should also be tested on actual fractures in cadavers, which would simulate the instability of broken bones in patients as well as the effects of surrounding tissues including tendons and ligaments. However, this would introduce several other variables regarding the fracture patterns, as well as the conditions of preserved cadaveric bones and soft tissues, which would make testing and interpretation difficult. Other standard diameters of surgical wires, and their corresponding fixator hole sizes, could also be tested, such as 0.7, 0.9, and 1.4 mm diameters, although we tested the 1.1 mm diameter which is the size most commonly used for finger fractures. The potential for adding more holes with decreased spacing should also be investigated, as the benefit of more wire positions might be counteracted by diminished wall thickness and thus lower stiffness. Lastly, although we tested flexion and lateral forces, we could also test torsional and axial forces, although these are rare under clinical conditions.

## 5. Conclusions

This novel 3D-printed PLA external finger fracture fixator device secures numerous surgical stainless-steel wires, maintains greater flexion stiffness and equivalent lateral stiffness compared to the wires even after sterilization with low-temperature HPGP, allows for static and dynamic fixation with compact, lightweight, and modular radiolucent components, and is both low-cost and customizable on demand, which may benefit under-resourced communities.

## Data Availability

The original data presented in the study are openly available in Figshare at https://doi.org/10.6084/m9.figshare.32980292.

## Supplementary Materials

The following supporting information can be downloaded at: https://www.mdpi.com/article/10.3390/bioengineering13090969/s1, Video S1: cadaver demonstration of dynamic fixation.

## Abbreviations

The following abbreviations are used in this manuscript:

PLA	Polylactic acid
PETG	Polyethylene terephthalate glycol
SSS	Surgical stainless-steel
PC	Polycarbonate
PEEK	Polyether ether ketone
